# 2-[(*E*)-({3-[(*E*)-(2-Hy­droxy­benzyl­idene)amino­meth­yl]-1,4-dioxaspiro­[4.5]decan-2-yl}meth­yl)imino­meth­yl]phenol

**DOI:** 10.1107/S1600536812029182

**Published:** 2012-06-30

**Authors:** Yan Jiang, Lili Wang, Jing Bian, Xiaoying Du, Xiaoqiang Sun

**Affiliations:** a Key Laboratory of Fine Chemical Engineering, Changzhou University, Changzhou 213164, Jiangsu, People’s Republic of China

## Abstract

In the title compound, C_24_H_28_N_2_O_4_, the dioxalane ring has an envelope conformation. The cyclo­hexane ring adopts a chair conformation. The dihedral angle between the benzene rings is 72.5 (3)°. The mol­ecular conformation is stabilized by two intra­molecular O—H⋯N hydrogen-bonding inter­actions with an *S*(6) graph-set motif. The crystal structure is stabilized by van der Waals inter­actions.

## Related literature

For the synthesis, see: Gan (2008 ▶). For standard bond lengths, see: Allen *et al.* (1987 ▶). For hydrogen-bond motifs, see: Bernstein *et al.* (1995 ▶). For ring conformations, see: Cremer & Pople (1975 ▶).
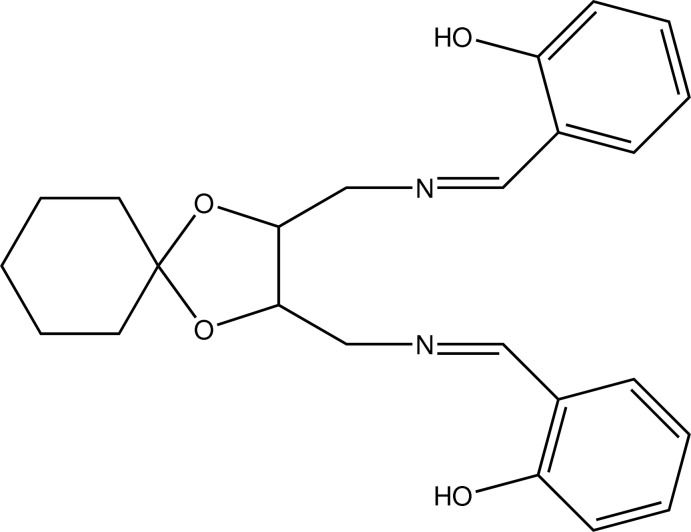


## Experimental

### 

#### Crystal data

C_24_H_28_N_2_O_4_
*M* *_r_* = 408.48
Monoclinic, 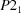

*a* = 5.7443 (8) Å
*b* = 21.558 (3) Å
*c* = 9.0075 (11) Å
β = 95.074 (6)°
*V* = 1111.1 (2) Å^3^
*Z* = 2
Mo *K*α radiation
μ = 0.08 mm^−1^
*T* = 296 K
0.20 × 0.18 × 0.15 mm

#### Data collection

Enraf–Nonius CAD-4 diffractometer
Absorption correction: ψ scan (North *et al.*, 1968 ▶) *T*_min_ = 0.984, *T*_max_ = 0.988
6560 measured reflections
2102 independent reflections
1522 reflections with *I* > 2σ(*I*)
*R*_int_ = 0.045
3 standard reflections every 200 reflections intensity decay: 1%

#### Refinement

*R*[*F* ^2^ > 2σ(*F* ^2^)] = 0.057
*wR*(*F* ^2^) = 0.169
*S* = 1.01
2102 reflections
285 parameters
13 restraints
H atoms treated by a mixture of independent and constrained refinement
Δρ_max_ = 0.57 e Å^−3^
Δρ_min_ = −0.33 e Å^−3^

### 

Data collection: *CAD-4 Software* (Enraf–Nonius, 1985 ▶); cell refinement: *CAD-4 Software*; data reduction: *XCAD4* (Harms & Wocadlo, 1995 ▶); program(s) used to solve structure: *SHELXS97* (Sheldrick, 2008 ▶); program(s) used to refine structure: *SHELXL97* (Sheldrick, 2008 ▶); molecular graphics: *SHELXTL* (Sheldrick, 2008 ▶); software used to prepare material for publication: *SHELXTL*.

## Supplementary Material

Crystal structure: contains datablock(s) I, global. DOI: 10.1107/S1600536812029182/bx2417sup1.cif

Structure factors: contains datablock(s) I. DOI: 10.1107/S1600536812029182/bx2417Isup2.hkl

Supplementary material file. DOI: 10.1107/S1600536812029182/bx2417Isup3.cml

Additional supplementary materials:  crystallographic information; 3D view; checkCIF report

## Figures and Tables

**Table 1:** Hydrogen-bond geometry (Å, °)

*D*—H⋯*A*	*D*—H	H⋯*A*	*D*⋯*A*	*D*—H⋯*A*
O3—H3⋯N2	0.82	1.91	2.561 (5)	135
O4—H4⋯N1	0.82 (8)	1.83 (8)	2.601 (6)	157 (8)

**Fig. 1.**
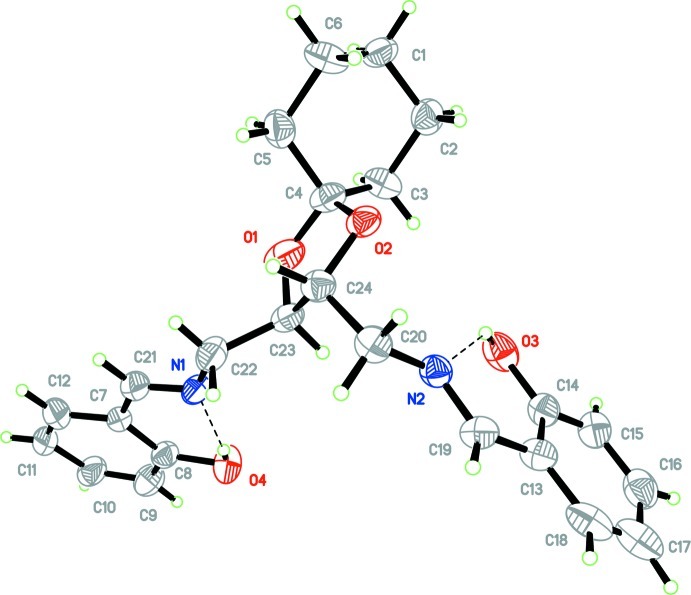
The molecular structure of (I), with the atom-numbering scheme. Displacement ellipsoids are drawn at the 50% probability level. The S(6) motifs is shows as dashed lines.

## supplementary crystallographic information

### Comment

Multidentate and chiral C*_2_*-symmetric ligands have attracted considerable interest, however, the number of chiral precursors available from nature is seriously limited. Gan, (2008) have reported some similar C2-symmetric tartaric acid derivatives which have ability to metal coordination and effect to catalytic Henry reaction.We have undertaken the X-ray crystal-structure determination of (I) in order to establish its molecular conformation and relative stereochemistry. We are not able to determine the absolute stereochemistry by X-ray methods. We report here the synthesis and the crystal structure of the title compound based on *L*-tartaric acid. The dioxalane ring has an envelope conformation.(Q2=0.291 (5)Å, φ_2_ = 286.4 (10)°. The cyclohexane ring adopt chair conformation (Q_T_= 0.560 (6) Å, θ= 175.4 (6)°, φ_2_ = 176.0 (8)° (Cremer & Pople, 1975). The dihedral angle between the two phenyl rings is 72.5 (3)°. The molecular conformation is stabilized by two intramolecular O—H···N hydrogen-bond interaction with graph-set motif *S*(6), (Bernstein *et al.*, 1995) .The crystal structure is stabilized by van der Waals interactions. The bond lengths and angles are within normal ranges (Allen *et al.*, 1987).

### Experimental

The title compound, (I) was prepared by a method reported by (Gan, 2008).To a solution of 2-hydroxybenzaldehyde(1.22 g, 10 mmol) in ethanol (15 ml), (2*S*,3S)-1,4-dioxaspiro[4.5]decane-2,3-diyldimethanamine(1 g, 5 mmol) dissolved in methanol(10 ml) was added. The mixture was refluxed for 2 h to complete the reaction and then cooled at room temperature. The compound was recrystallized from ethanol to afford a yellow solid (1.3 g, 63.7% yield, m.p. 360.45–361.25 K). Single crystal suitable for X-ray diffraction were also obtained by evaporation of an ethanol solution. The crystals were obtained by dissolving (I) (0.5 g, 1.22 mmol) in ethanol (25 ml) and evaporating the solvent slowly at room temperature for about 7 d.

### Refinement

All H atoms were positioned geometrically and constrained to ride on their parent atoms, with C—H = 0.93 Å for aromatic H. Other H atoms were positioned geometrically and refined using a riding model, with C—H = 0.97 Å for alkyl H, with *U*_iso_(H) = *xU*_eq_(C), where *x* = 1.2 for aromatic H, and *x* = 1.5 for other H. In the absence of anomalous scatterers the absolute configuration could not be determined and therefore Friedel pairs were merged.

### Crystal data

**Table:**

C_24_H_28_N_2_O_4_	*F*(000) = 436
*M**_r_* = 408.48	*D*_x_ = 1.221 Mg m^−^^3^
Monoclinic, *P*2_1_	Mo *K*α radiation, λ = 0.71073 Å
Hall symbol: P 2yb	Cell parameters from 1658 reflections
*a* = 5.7443 (8) Å	θ = 2.3–20.6°
*b* = 21.558 (3) Å	µ = 0.08 mm^−^^1^
*c* = 9.0075 (11) Å	*T* = 296 K
β = 95.074 (6)°	Block, colourless
*V* = 1111.1 (2) Å^3^	0.20 × 0.18 × 0.15 mm
*Z* = 2	

### Data collection

**Table:**

Enraf–Nonius CAD-4 diffractometer	1522 reflections with *I* > 2σ(*I*)
Radiation source: fine-focus sealed tube	*R*_int_ = 0.045
Graphite monochromator	θ_max_ = 25.5°, θ_min_ = 1.9°
ω/2θ scans	*h* = −6→6
Absorption correction: ψ scan (North *et al.*, 1968)	*k* = −26→25
*T*_min_ = 0.984, *T*_max_ = 0.988	*l* = −9→10
6560 measured reflections	3 standard reflections every 200 reflections
2102 independent reflections	intensity decay: 1%

### Refinement

**Table:**

Refinement on *F*^2^	Primary atom site location: structure-invariant direct methods
Least-squares matrix: full	Secondary atom site location: difference Fourier map
*R*[*F*^2^ > 2σ(*F*^2^)] = 0.057	Hydrogen site location: inferred from neighbouring sites
*wR*(*F*^2^) = 0.169	H atoms treated by a mixture of independent and constrained refinement
*S* = 1.01	*w* = 1/[σ^2^(*F*_o_^2^) + (0.1156*P*)^2^] where *P* = (*F*_o_^2^ + 2*F*_c_^2^)/3
2102 reflections	(Δ/σ)_max_ = 0.002
285 parameters	Δρ_max_ = 0.57 e Å^−^^3^
13 restraints	Δρ_min_ = −0.33 e Å^−^^3^

### Special details

**Table:**

Geometry. All e.s.d.'s (except the e.s.d. in the dihedral angle between two l.s. planes) are estimated using the full covariance matrix. The cell e.s.d.'s are taken into account individually in the estimation of e.s.d.'s in distances, angles and torsion angles; correlations between e.s.d.'s in cell parameters are only used when they are defined by crystal symmetry. An approximate (isotropic) treatment of cell e.s.d.'s is used for estimating e.s.d.'s involving l.s. planes.
Refinement. Refinement of *F*^2^ against ALL reflections. The weighted *R*-factor *wR* and goodness of fit *S* are based on *F*^2^, conventional *R*-factors *R* are based on *F*, with *F* set to zero for negative *F*^2^. The threshold expression of *F*^2^ > σ(*F*^2^) is used only for calculating *R*-factors(gt) *etc*. and is not relevant to the choice of reflections for refinement. *R*-factors based on *F*^2^ are statistically about twice as large as those based on *F*, and *R*- factors based on ALL data will be even larger.

### Fractional atomic coordinates and isotropic or equivalent isotropic displacement parameters (Å^2^)

**Table:**

	*x*	*y*	*z*	*U*_iso_*/*U*_eq_	
O2	0.7032 (5)	0.0442 (2)	0.2755 (3)	0.0579 (8)	
N1	0.4452 (7)	0.19135 (19)	0.6142 (4)	0.0587 (10)	
N2	0.7647 (7)	−0.01611 (17)	0.5564 (4)	0.0515 (9)	
O3	0.3731 (6)	−0.0730 (2)	0.5143 (5)	0.0793 (11)	
H3	0.4865	−0.0579	0.4784	0.119*	
O4	0.1145 (9)	0.1775 (2)	0.7908 (5)	0.0814 (12)	
C7	0.2202 (10)	0.2762 (2)	0.6925 (5)	0.0613 (13)	
C19	0.8210 (8)	−0.0504 (2)	0.6661 (5)	0.0573 (12)	
H19	0.9640	−0.0443	0.7209	0.069*	
C24	0.7982 (8)	0.0768 (2)	0.4043 (5)	0.0536 (11)	
H24	0.9080	0.1086	0.3760	0.064*	
C23	0.5846 (8)	0.1074 (2)	0.4606 (5)	0.0560 (11)	
H23	0.5118	0.0790	0.5278	0.067*	
C14	0.4466 (9)	−0.1094 (2)	0.6303 (6)	0.0597 (12)	
C13	0.6671 (8)	−0.0998 (2)	0.7097 (5)	0.0587 (12)	
C8	0.0807 (9)	0.2405 (2)	0.7772 (5)	0.0601 (13)	
C20	0.9224 (7)	0.0312 (2)	0.5111 (5)	0.0584 (12)	
H20A	1.0488	0.0118	0.4636	0.070*	
H20B	0.9898	0.0533	0.5985	0.070*	
C21	0.4048 (11)	0.2486 (3)	0.6123 (6)	0.0667 (14)	
H21	0.4955	0.2744	0.5579	0.080*	
C22	0.6410 (11)	0.1678 (3)	0.5390 (7)	0.0675 (14)	
C4	0.5053 (8)	0.0777 (3)	0.2129 (5)	0.0636 (12)	
C1	0.4141 (10)	0.0380 (3)	−0.0967 (5)	0.0721 (14)	
H1A	0.4493	0.0136	−0.1824	0.087*	
H1B	0.2772	0.0630	−0.1254	0.087*	
C10	−0.1293 (12)	0.3299 (3)	0.8382 (8)	0.0847 (19)	
H10	−0.2521	0.3474	0.8844	0.102*	
C2	0.3642 (11)	−0.0043 (3)	0.0280 (6)	0.0752 (16)	
H2A	0.4974	−0.0312	0.0527	0.090*	
H2B	0.2301	−0.0301	−0.0024	0.090*	
C16	0.3780 (13)	−0.1954 (3)	0.7878 (8)	0.0859 (18)	
H16	0.2854	−0.2288	0.8112	0.103*	
C12	0.1847 (16)	0.3400 (3)	0.6869 (7)	0.096 (2)	
H12	0.2806	0.3647	0.6336	0.115*	
C15	0.3060 (11)	−0.1579 (3)	0.6720 (7)	0.0732 (15)	
H15	0.1612	−0.1646	0.6198	0.088*	
C6	0.6157 (11)	0.0792 (3)	−0.0503 (6)	0.0833 (18)	
H6A	0.6461	0.1062	−0.1326	0.100*	
H6B	0.7540	0.0541	−0.0260	0.100*	
C9	−0.0925 (11)	0.2673 (3)	0.8512 (7)	0.0775 (16)	
H9	−0.1835	0.2433	0.9094	0.093*	
C18	0.7323 (12)	−0.1387 (4)	0.8281 (7)	0.0866 (19)	
H18	0.8785	−0.1336	0.8798	0.104*	
C3	0.3153 (9)	0.0337 (3)	0.1633 (6)	0.0712 (14)	
H3A	0.1719	0.0569	0.1403	0.085*	
H3B	0.2906	0.0057	0.2447	0.085*	
C5	0.5689 (10)	0.1184 (3)	0.0836 (6)	0.0738 (15)	
H5A	0.4417	0.1469	0.0562	0.089*	
H5B	0.7069	0.1426	0.1149	0.089*	
C11	0.0057 (15)	0.3675 (3)	0.7606 (7)	0.094 (2)	
H11	−0.0195	0.4101	0.7565	0.113*	
C17	0.5884 (14)	−0.1845 (4)	0.8717 (8)	0.102 (2)	
H17	0.6310	−0.2080	0.9564	0.122*	
H22A	0.682 (10)	0.196 (3)	0.479 (7)	0.075 (18)*	
H22B	0.802 (9)	0.158 (2)	0.614 (6)	0.067 (15)*	
H4	0.237 (13)	0.175 (4)	0.752 (9)	0.10 (3)*	
O1	0.4339 (7)	0.1163 (3)	0.3301 (4)	0.0890 (12)	

### Atomic displacement parameters (Å^2^)

**Table:**

	*U*^11^	*U*^22^	*U*^33^	*U*^12^	*U*^13^	*U*^23^
O2	0.0607 (18)	0.0631 (18)	0.0497 (17)	0.0170 (16)	0.0035 (13)	−0.0074 (15)
N1	0.069 (2)	0.052 (2)	0.055 (2)	0.007 (2)	0.0034 (18)	−0.0128 (18)
N2	0.050 (2)	0.052 (2)	0.051 (2)	0.0082 (18)	0.0011 (16)	0.0039 (17)
O3	0.063 (2)	0.080 (2)	0.091 (3)	0.000 (2)	−0.017 (2)	0.024 (2)
O4	0.080 (3)	0.057 (2)	0.110 (3)	0.001 (2)	0.022 (2)	−0.005 (2)
C7	0.089 (3)	0.051 (3)	0.043 (2)	0.016 (3)	0.004 (2)	−0.001 (2)
C19	0.049 (2)	0.072 (3)	0.051 (3)	0.012 (2)	0.005 (2)	−0.002 (2)
C24	0.049 (2)	0.061 (3)	0.051 (2)	0.006 (2)	0.0059 (18)	−0.003 (2)
C23	0.054 (2)	0.065 (3)	0.047 (2)	0.012 (2)	−0.0030 (18)	−0.014 (2)
C14	0.059 (3)	0.056 (3)	0.065 (3)	0.019 (2)	0.011 (2)	0.003 (2)
C13	0.053 (3)	0.071 (3)	0.053 (2)	0.017 (2)	0.010 (2)	0.005 (2)
C8	0.063 (3)	0.057 (3)	0.059 (3)	0.000 (2)	−0.004 (2)	−0.010 (2)
C20	0.047 (2)	0.065 (3)	0.062 (3)	0.004 (2)	−0.001 (2)	0.003 (2)
C21	0.093 (4)	0.054 (3)	0.055 (3)	0.002 (3)	0.017 (3)	0.000 (2)
C22	0.078 (4)	0.055 (3)	0.073 (4)	−0.001 (3)	0.021 (3)	−0.012 (3)
C4	0.059 (3)	0.081 (3)	0.049 (2)	0.021 (2)	−0.0022 (19)	−0.013 (2)
C1	0.090 (4)	0.077 (3)	0.050 (3)	0.010 (3)	0.003 (2)	−0.014 (3)
C10	0.078 (4)	0.091 (4)	0.082 (4)	0.032 (4)	−0.008 (3)	−0.024 (4)
C2	0.083 (4)	0.065 (3)	0.075 (4)	−0.010 (3)	−0.008 (3)	−0.007 (3)
C16	0.099 (5)	0.073 (4)	0.090 (4)	0.001 (4)	0.033 (4)	0.012 (3)
C12	0.152 (7)	0.070 (4)	0.069 (4)	0.035 (4)	0.027 (4)	0.015 (3)
C15	0.070 (3)	0.068 (3)	0.084 (4)	0.002 (3)	0.017 (3)	0.002 (3)
C6	0.087 (4)	0.106 (5)	0.058 (3)	−0.009 (4)	0.018 (3)	0.021 (3)
C9	0.068 (3)	0.079 (4)	0.087 (4)	−0.003 (3)	0.014 (3)	−0.016 (3)
C18	0.077 (4)	0.109 (5)	0.073 (4)	0.016 (4)	0.004 (3)	0.039 (4)
C3	0.062 (3)	0.091 (4)	0.060 (3)	−0.005 (3)	0.005 (2)	0.016 (3)
C5	0.089 (4)	0.054 (3)	0.074 (4)	−0.006 (3)	−0.014 (3)	0.004 (3)
C11	0.149 (7)	0.071 (4)	0.064 (3)	0.045 (4)	0.020 (4)	0.002 (3)
C17	0.098 (5)	0.120 (6)	0.089 (5)	0.015 (5)	0.021 (4)	0.048 (4)
O1	0.084 (2)	0.111 (3)	0.067 (2)	0.045 (2)	−0.0180 (17)	−0.0291 (19)

### Geometric parameters (Å, º)

**Table:**

O2—C4	1.420 (6)	C4—C3	1.485 (8)
O2—C24	1.424 (6)	C4—C5	1.528 (8)
N1—C21	1.257 (7)	C1—C6	1.490 (8)
N1—C22	1.455 (7)	C1—C2	1.494 (8)
N2—C19	1.253 (6)	C1—H1A	0.9700
N2—C20	1.447 (6)	C1—H1B	0.9700
O3—C14	1.344 (7)	C10—C11	1.357 (10)
O3—H3	0.8200	C10—C9	1.370 (9)
O4—C8	1.378 (8)	C10—H10	0.9300
O4—H4	0.82 (7)	C2—C3	1.515 (8)
C7—C8	1.387 (8)	C2—H2A	0.9700
C7—C12	1.389 (8)	C2—H2B	0.9700
C7—C21	1.461 (8)	C16—C15	1.355 (9)
C19—C13	1.460 (7)	C16—C17	1.388 (11)
C19—H19	0.9300	C16—H16	0.9300
C24—C20	1.509 (7)	C12—C11	1.404 (10)
C24—C23	1.519 (6)	C12—H12	0.9300
C24—H24	0.9800	C15—H15	0.9300
C23—O1	1.410 (6)	C6—C5	1.516 (8)
C23—C22	1.503 (7)	C6—H6A	0.9700
C23—H23	0.9800	C6—H6B	0.9700
C14—C15	1.393 (7)	C9—H9	0.9300
C14—C13	1.414 (7)	C18—C17	1.367 (10)
C13—C18	1.383 (8)	C18—H18	0.9300
C8—C9	1.373 (8)	C3—H3A	0.9700
C20—H20A	0.9700	C3—H3B	0.9700
C20—H20B	0.9700	C5—H5A	0.9700
C21—H21	0.9300	C5—H5B	0.9700
C22—H22A	0.86 (6)	C11—H11	0.9300
C22—H22B	1.12 (6)	C17—H17	0.9300
C4—O1	1.434 (6)		
			
C4—O2—C24	107.9 (4)	C6—C1—H1A	109.6
C21—N1—C22	119.1 (5)	C2—C1—H1A	109.6
C19—N2—C20	121.0 (4)	C6—C1—H1B	109.6
C14—O3—H3	109.5	C2—C1—H1B	109.6
C8—O4—H4	98 (6)	H1A—C1—H1B	108.1
C8—C7—C12	118.6 (6)	C11—C10—C9	122.8 (7)
C8—C7—C21	121.7 (4)	C11—C10—H10	118.6
C12—C7—C21	119.7 (6)	C9—C10—H10	118.6
N2—C19—C13	121.5 (4)	C1—C2—C3	109.6 (5)
N2—C19—H19	119.3	C1—C2—H2A	109.7
C13—C19—H19	119.3	C3—C2—H2A	109.7
O2—C24—C20	108.8 (4)	C1—C2—H2B	109.7
O2—C24—C23	102.9 (3)	C3—C2—H2B	109.7
C20—C24—C23	114.8 (4)	H2A—C2—H2B	108.2
O2—C24—H24	110.0	C15—C16—C17	120.7 (6)
C20—C24—H24	110.0	C15—C16—H16	119.7
C23—C24—H24	110.0	C17—C16—H16	119.7
O1—C23—C22	111.3 (5)	C7—C12—C11	120.8 (7)
O1—C23—C24	103.6 (4)	C7—C12—H12	119.6
C22—C23—C24	112.7 (4)	C11—C12—H12	119.6
O1—C23—H23	109.7	C16—C15—C14	120.7 (6)
C22—C23—H23	109.7	C16—C15—H15	119.6
C24—C23—H23	109.7	C14—C15—H15	119.6
O3—C14—C15	119.9 (5)	C1—C6—C5	111.6 (5)
O3—C14—C13	120.9 (5)	C1—C6—H6A	109.3
C15—C14—C13	119.2 (5)	C5—C6—H6A	109.3
C18—C13—C14	118.1 (5)	C1—C6—H6B	109.3
C18—C13—C19	121.4 (5)	C5—C6—H6B	109.3
C14—C13—C19	120.5 (4)	H6A—C6—H6B	108.0
C9—C8—O4	118.3 (6)	C10—C9—C8	119.1 (7)
C9—C8—C7	120.8 (5)	C10—C9—H9	120.5
O4—C8—C7	120.9 (5)	C8—C9—H9	120.5
N2—C20—C24	111.5 (3)	C17—C18—C13	122.0 (6)
N2—C20—H20A	109.3	C17—C18—H18	119.0
C24—C20—H20A	109.3	C13—C18—H18	119.0
N2—C20—H20B	109.3	C4—C3—C2	113.8 (4)
C24—C20—H20B	109.3	C4—C3—H3A	108.8
H20A—C20—H20B	108.0	C2—C3—H3A	108.8
N1—C21—C7	122.3 (5)	C4—C3—H3B	108.8
N1—C21—H21	118.8	C2—C3—H3B	108.8
C7—C21—H21	118.8	H3A—C3—H3B	107.7
N1—C22—C23	112.2 (5)	C6—C5—C4	110.9 (4)
N1—C22—H22A	108 (4)	C6—C5—H5A	109.5
C23—C22—H22A	112 (4)	C4—C5—H5A	109.5
N1—C22—H22B	115 (3)	C6—C5—H5B	109.5
C23—C22—H22B	105 (3)	C4—C5—H5B	109.5
H22A—C22—H22B	105 (5)	H5A—C5—H5B	108.0
O2—C4—O1	105.9 (3)	C10—C11—C12	117.8 (6)
O2—C4—C3	109.6 (5)	C10—C11—H11	121.1
O1—C4—C3	110.0 (5)	C12—C11—H11	121.1
O2—C4—C5	110.9 (4)	C18—C17—C16	119.0 (6)
O1—C4—C5	109.3 (5)	C18—C17—H17	120.5
C3—C4—C5	110.9 (4)	C16—C17—H17	120.5
C6—C1—C2	110.4 (4)	C23—O1—C4	109.9 (4)
			
C20—N2—C19—C13	−177.8 (4)	C6—C1—C2—C3	57.8 (7)
C4—O2—C24—C20	152.8 (4)	C8—C7—C12—C11	−2.3 (10)
C4—O2—C24—C23	30.6 (5)	C21—C7—C12—C11	178.8 (6)
O2—C24—C23—O1	−29.2 (5)	C17—C16—C15—C14	3.0 (9)
C20—C24—C23—O1	−147.3 (5)	O3—C14—C15—C16	179.4 (6)
O2—C24—C23—C22	−149.6 (5)	C13—C14—C15—C16	0.4 (8)
C20—C24—C23—C22	92.2 (6)	C2—C1—C6—C5	−59.3 (7)
O3—C14—C13—C18	179.9 (5)	C11—C10—C9—C8	−3.3 (10)
C15—C14—C13—C18	−1.2 (8)	O4—C8—C9—C10	−179.6 (6)
O3—C14—C13—C19	−0.6 (7)	C7—C8—C9—C10	1.3 (9)
C15—C14—C13—C19	178.3 (4)	C14—C13—C18—C17	−1.6 (10)
N2—C19—C13—C18	178.1 (5)	C19—C13—C18—C17	178.9 (6)
N2—C19—C13—C14	−1.3 (7)	O2—C4—C3—C2	−70.3 (5)
C12—C7—C8—C9	1.4 (8)	O1—C4—C3—C2	173.6 (5)
C21—C7—C8—C9	−179.7 (5)	C5—C4—C3—C2	52.5 (6)
C12—C7—C8—O4	−177.6 (5)	C1—C2—C3—C4	−55.8 (6)
C21—C7—C8—O4	1.2 (8)	C1—C6—C5—C4	55.5 (6)
C19—N2—C20—C24	−165.4 (4)	O2—C4—C5—C6	70.8 (6)
O2—C24—C20—N2	−59.9 (5)	O1—C4—C5—C6	−172.8 (4)
C23—C24—C20—N2	54.8 (5)	C3—C4—C5—C6	−51.3 (6)
C22—N1—C21—C7	−176.3 (5)	C9—C10—C11—C12	2.4 (11)
C8—C7—C21—N1	0.8 (8)	C7—C12—C11—C10	0.5 (11)
C12—C7—C21—N1	179.6 (6)	C13—C18—C17—C16	5.0 (11)
C21—N1—C22—C23	−141.1 (5)	C15—C16—C17—C18	−5.7 (11)
O1—C23—C22—N1	73.8 (6)	C22—C23—O1—C4	139.1 (5)
C24—C23—C22—N1	−170.2 (4)	C24—C23—O1—C4	17.8 (6)
C24—O2—C4—O1	−20.3 (6)	O2—C4—O1—C23	0.5 (7)
C24—O2—C4—C3	−139.0 (4)	C3—C4—O1—C23	118.9 (5)
C24—O2—C4—C5	98.2 (4)	C5—C4—O1—C23	−119.1 (5)

### Hydrogen-bond geometry (Å, º)

**Table:**

*D*—H···*A*	*D*—H	H···*A*	*D*···*A*	*D*—H···*A*
O3—H3···N2	0.82	1.91	2.561 (5)	135
O4—H4···N1	0.82 (8)	1.83 (8)	2.601 (6)	157 (8)
